# Supplementary material for: Multitrophic interaction networks mediate biodiversity effects on ecosystem multifunctionality
Source: Nat Commun. 2026 Jul 2;17:5787. doi: 10.1038/s41467-026-75046-0 (PMC13328580; doi:10.1038/s41467-026-75046-0)
Supplement: Supplementary file 2 — Description of Additional Supplementary Files [file 41467_2026_75046_MOESM2_ESM.pdf]

Description of Additional Supplementary Files

File name: Supplementary Code 1
Description: Code used for the analysis, including necessary variable transformation and model selection as also described in the methods.
