# Supplementary material for: Multitrophic interaction networks mediate biodiversity effects on ecosystem multifunctionality
Source: Nat Commun. 2026 Jul 2;17:5787. doi: 10.1038/s41467-026-75046-0 (PMC13328580; doi:10.1038/s41467-026-75046-0)
Supplement: Supplementary file 4 — Reporting Summary [file 41467_2026_75046_MOESM4_ESM.pdf]

nature portfolio

Corresponding author(s): Georg Albert

Last updated by author(s): May 20, 2026

Reporting Summary

Nature Portfolio wishes to improve the reproducibility of the work that we publish. This form provides structure for consistency and transparency in reporting. For further information on Nature Portfolio policies, see our Editorial Policies and the Editorial Policy Checklist.

Statistics

For all statistical analyses, confirm that the following items are present in the figure legend, table legend, main text, or Methods section.

n/a	Confirmed	
☐	☒	The exact sample size (*n*) for each experimental group/condition, given as a discrete number and unit of measurement
☐	☒	A statement on whether measurements were taken from distinct samples or whether the same sample was measured repeatedly
☐	☒	The statistical test(s) used AND whether they are one- or two-sided<br>*Only common tests should be described solely by name; describe more complex techniques in the Methods section.*
☐	☒	A description of all covariates tested
☐	☒	A description of any assumptions or corrections, such as tests of normality and adjustment for multiple comparisons
☐	☒	A full description of the statistical parameters including central tendency (e.g. means) or other basic estimates (e.g. regression coefficient) AND variation (e.g. standard deviation) or associated estimates of uncertainty (e.g. confidence intervals)
☐	☒	For null hypothesis testing, the test statistic (e.g. *F*, *t*, *r*) with confidence intervals, effect sizes, degrees of freedom and *P* value noted<br>*Give P values as exact values whenever suitable.*
☒	☐	For Bayesian analysis, information on the choice of priors and Markov chain Monte Carlo settings
☒	☐	For hierarchical and complex designs, identification of the appropriate level for tests and full reporting of outcomes
☐	☒	Estimates of effect sizes (e.g. Cohen's *d*, Pearson's *r*), indicating how they were calculated

Our web collection on statistics for biologists contains articles on many of the points above.

Software and code

Policy information about availability of computer code

Data collection	n/a
Data analysis	All analyses were conducted with R version 4.3.3 (packages: lavaan, piecewiseSEM, lme4, missForest, LambertW)

For manuscripts utilizing custom algorithms or software that are central to the research but not yet described in published literature, software must be made available to editors and reviewers. We strongly encourage code deposition in a community repository (e.g. GitHub). See the Nature Portfolio guidelines for submitting code & software for further information.

Data

Policy information about availability of data

All manuscripts must include a data availability statement. This statement should provide the following information, where applicable:

- Accession codes, unique identifiers, or web links for publicly available datasets
- A description of any restrictions on data availability
- For clinical datasets or third party data, please ensure that the statement adheres to our policy

The data underlying the analyses presented in this study are available from figshare at https://doi.org/10.6084/m9.figshare.27925374. Source data are provided with this paper.

Research involving human participants, their data, or biological material

Policy information about studies with human participants or human data. See also policy information about sex, gender (identity/presentation), and sexual orientation and race, ethnicity and racism.

Reporting on sex and gender	n/a
Reporting on race, ethnicity, or other socially relevant groupings	n/a
Population characteristics	n/a
Recruitment	n/a
Ethics oversight	n/a

Note that full information on the approval of the study protocol must also be provided in the manuscript.

Field-specific reporting

Please select the one below that is the best fit for your research. If you are not sure, read the appropriate sections before making your selection.

☐ Life sciences ☐ Behavioural & social sciences ☒ Ecological, evolutionary & environmental sciences

For a reference copy of the document with all sections, see nature.com/documents/nr-reporting-summary-flat.pdf

Ecological, evolutionary & environmental sciences study design

All studies must disclose on these points even when the disclosure is negative.

Study description	The study uses species interaction data collected from a large-scale forest biodiversity experiment in China, the BEF-China experiment, to investigate how the structure of species interaction networks (network size, niche overlap, interaction evenness, linkage density) mediate effects of tree species richness on ecosystem multifunctionality.
Research sample	We treated experimental plots (manipulation of tree species richness) with a sufficient coverage of interaction and ecosystem functioning data as research samples, yielding a total of 69 plots. The study integrates a wide range of interaction and ecosystem functioning data that has been previously published. More details and relvant references can be found in the manuscript.
Sampling strategy	This study synthesizes data collected for previous research projects. To assure that the data is comparable between plots, we used resampling approaches to standardize sampling efforts. We used a total of 69 plots (i.e. sample units) for our analysis, which is sufficient for the structural equation models presented, with common recommendations for a minimum number of samples per path ranging from 1 to 5.
Data collection	Data on 34 ecosystem functions and 11 interaction networks was collected by experts on their respective taxonomic groups and ecosystem functions, many of which are co-authors of the study. The methods range from visual inspections (ant-Hemiptera interactions) to metabarcoding approaches (e.g. soil fungi) and fluorometric assays (litter enzyme activity). An extensive overview is provided in the supplementary information.
Timing and spatial scale	The data was collected between 2014-2019, with only one ecosystem function being sampled more recently (parasitism rates on Lepidoptera larvae in 2021-2022). The study covers both experimental sites of the experiment. An extensive overview is provided in the supplementary information.
Data exclusions	To be able to construct meaningful interaction networks for interaction networks including trees, we excluded all monoculture plots (i.e. plots with a single tree species).
Reproducibility	Detailed description of the methods are provided, data is available, and the analyses were performed with the freely available programming language R. While many other tree diversity experiments exists, we are not aware of any that has produced the wide range of data synthesized in this work.
Randomization	In our tree biodiversity experiment, tree species richness levels and tree compositions were randomly assigned to plots. Within plots, planting positions were randomly assigned.
Blinding	Blinding was not applicable because investigators were responsible for field sampling following predefined and standardized sampling protocols without subjective assessments. Analyses were based on objective measurements of interaction and ecosystem functioning data.

Did the study involve field work? ☒ Yes ☐ No

Field work, collection and transport

Field conditions	The BEF-China experiment is located in the subtropics of China with a mean annual temperature and precipitation of 16.7 °C and 1821 mm, respectively.
Location	The experiment is located in Xingangshan, Jiangxi Province (29°05'00''–29°07'43''N,117°54'19''–117°55'53''E).
Access & import/export	The BEF-China experiment was established with permission of the local authorities in sites which were previously occupied by economic tree monoculture plantations. The access to the experiment is based on small paths through the hills with minimal impact on trees. Our study conforms to the legal requirements of the People's Republic of China. Access to the experiment is granted through the land-renting contract with the Xingangshan Forestry Co. Ltd., Dexing, Jiangxi Province, China (04.11.2008). Sharing samples and data is provided through an agreement with the Institute of Botany, Chinese Academy of Sciences, Beijing, 10093 (05.2010).
Disturbance	This study did not cause any environmental disturbance. To avoid negative impacts of sampling, all research endeavors are coordinated by the steering committee of the BEF-China experiment.

Reporting for specific materials, systems and methods

We require information from authors about some types of materials, experimental systems and methods used in many studies. Here, indicate whether each material, system or method listed is relevant to your study. If you are not sure if a list item applies to your research, read the appropriate section before selecting a response.

Materials & experimental systems

n/a	Involved in the study	
☒	☐	Antibodies
☒	☐	Eukaryotic cell lines
☒	☐	Palaeontology and archaeology
☐	☒	Animals and other organisms
☒	☐	Clinical data
☒	☐	Dual use research of concern
☐	☒	Plants

Methods

n/a	Involved in the study	
☒	☐	ChIP-seq
☒	☐	Flow cytometry
☒	☐	MRI-based neuroimaging

Animals and other research organisms

Policy information about studies involving animals; ARRIVE guidelines recommended for reporting animal research, and Sex and Gender in Research

Laboratory animals	The study did not involve laboratory animals
Wild animals	Sampled organisms include arthropods, microorganisms, and soil fauna. A total of 4,028 species (including MOTUs) were collected.
Reporting on sex	n/a
Field-collected samples	Samples were stored according to common practice (e.g. arthropods in ethanol, if not assessed visually).
Ethics oversight	No specific ethical approval was necessary for the sampled species, and sampling was carried out with permission of the BEF-China steering committee and in accordance with local authorities

Note that full information on the approval of the study protocol must also be provided in the manuscript.

Plants

Seed stocks	Tree seeds were sourced locally prior to the establishment of the experiment (2008) and raised in local nurseries.
Novel plant genotypes	n/a
Authentication	n/a
