# Supplementary material for: Multitrophic interaction networks mediate biodiversity effects on ecosystem multifunctionality
Source: Nat Commun. 2026 Jul 2;17:5787. doi: 10.1038/s41467-026-75046-0 (PMC13328580; doi:10.1038/s41467-026-75046-0)
Supplement: Supplementary file 5 — Transparent Peer Review file [file 41467_2026_75046_MOESM5_ESM.pdf]

nature portfolio

Peer Review File

Multitrophic interaction networks mediate biodiversity effects on ecosystem multifunctionality

Corresponding Author: Dr Georg Albert

This file contains all reviewer reports in order by version, followed by all author rebuttals in order by version.

Version 0:

Reviewer comments:

Reviewer #1

(Remarks to the Author)
Albert et al. present a compelling and innovative study examining how tree diversity influences ecosystem multifunctionality through the structure of mutualistic and antagonistic interaction networks. The manuscript is well written, methodologically ambitious, and makes an important contribution by integrating concepts of biodiversity–ecosystem functioning with ecological network approaches. The results are clearly presented and supported by strong visualisations. However, I have some suggestions that I believe would improve the clarity, conceptual framing, and robustness of the analyses.

Major Comments
1. Conceptual framing and predictions
The results highlight a set of direct and indirect effects of tree richness on network structure and ecosystem multifunctionality. However, the introduction currently offers limited conceptual background on how and why tree diversity - particularly as primary producers - should influence ecological interactions, network topology, and multifunctionality. Strengthening this section with clearer mechanistic reasoning and explicit hypotheses or predictions would better guide the reader and help position the study within existing theory.

2. Joint effects of interaction types
The separate evaluation of antagonistic and mutualistic networks is interesting and informative. Nonetheless, it is not clear why the authors chose not to assess a combined or integrative effect of these networks. Ecological processes often emerge from the interplay among multiple interaction types, and exploring whether or how antagonistic and mutualistic networks jointly mediate biodiversity effects could provide additional insights. At minimum, the rationale for analysing them separately should be clarified.

3. Accounting for hierarchical structure and temporal replication
The experimental design appears hierarchical (plots nested within sites), and sampling spans multiple years (2014–2019). Such structure raises concerns about non-independence among observations. A mixed modelling framework, either linear or generalized, depending on the response variables, would seem more appropriate for handling random site, plot, and temporal effects. In addition, a piecewise SEM approach (e.g., Lefcheck 2016) could offer greater flexibility for integrating causal paths while accounting for this nested design. Clarifying how these dependencies are addressed would enhance confidence in the statistical inference.

4. Scope and generality of inference
The experiment includes only two sites, each with multiple subplots. This limited spatial replication constrains the generalizability of the findings. The authors should more clearly discuss the degree to which the observed patterns can be extrapolated beyond the study system, and under what ecological or environmental conditions the proposed mechanisms are expected to hold.

Minor Suggestion
5. Data transparency and reproducibility
To facilitate reproducibility and improve data accessibility, I recommend including a README file that explains the dataset(s)' structure, variable definitions, and the workflow used for the analyses. This would greatly aid readers attempting to understand or reuse the data and code.

Reviewer #2

(Remarks to the Author)
In this study, Albert and co-authors investigated how multritophic interaction networks mediate effects of manipulated tree diversity on ecosystem multifunctionality. Specifically, they hypothesize that because many ecosystem functions are driven by species interactions, and these species interactions depend on (tree or multitrophic) diversity, species interactions should mediate biodiversity-EMF relationships. They tested these predictions based on a large tree biodiversity experiment, where interaction networks as well as various ecosystem function rates were measured.
In general, I am impressed by this study, which relies on a large experiment, huge amounts of data and a nice set of original ideas. At the same time, I do have some concerns which currently still hamper this study.
First, when looking at the functions that were included to study ecosystem multifunctionality, the majority of the functions were either (proxies of) biomass stocks, or of energy flows. This means that EMF is generally highest in ecosystems with high biomass stocks and flows. So far so good. But there were exceptions of functions that really not fitted into this, and thereby blur the interpretation of the findings. For example, variables like N and P resorption, as well as erosion control are not examples of biomass stocks or flows (even if they are to some extent related, they are fundamentally different), so I do not think it is wise to include these functions when quantifying ecosystem multifunctionality.
Second, when looking at the more detailed results (on individual ecosystem functions) that are a bit hidden in Supplementary Table 7, I found that some observed relationships were rather surprising. For example, spider-prey interactions seem rather predictive of fungal fruitbody numbers, while understory biomass was most related to spider-prey interactions (based on spiders that were active in aboveground tree branches). It would be good to discuss the presence of unexpected relationships a bit, even though this discussion should mostly focus on a meta-level, as one can't possibly discuss everything.
The authors also tried to sell their paper a bit as informing conservation. For example, at the end of the abstract, the authors write "These findings emphasize that to effectively safeguard ecosystem service provisioning, ecosystem management and biodiversity conservation not only need to account for biodiversity changes at multiple trophic levels, but also explicitly for how species interact among each other." It is quite popular in ecological papers to claim that findings have applied value for nature management, but a statement like this feels a bit empty if not any concrete examples (also not in the main text) are given. So if the authors believe in this statement, then I would also expect them to really work this argument out, and give concrete examples (so: how can e.g. forest managers adjust their management in order to stimulate species interactions that promote ecosystem multifunctionality? And is it likely that they can be convinced to do so, or would they have other priorities, even if they do care about biodiversity conservation). Otherwise, it would be better to remove sentences like these.
I also have several more specific comments, listed below.

L120-122: the high number of dependent clauses makes this sentence rather difficult to understand
L129-130: "limits our ability to accurately predict the consequences of global biodiversity": I find the focus on global biodiversity a bit strange, because species interactions seem mostly relevant at local scales (plants/animals need to be sufficiently close to each other to interact), but less so at very large (or even global) scales.
Line 135: "to accurately predict the consequences of global biodiversity": would be nice to add an argument on why this is the case
L 139: "biodiversity" should be replaced by "biodiversity-ecosystem functioning"
L 144-146: I found this mechanism not so well explained yet. What is meant by "complementarity in species interactions"? Can you give an example? For example, are competitive and trophic interactions complementary to each other? And what does complementarity mean in this context?
L 151-152: it would be good to explain a bit more what is meant by "the evenness distribution of species interactions (i.e. interaction evenness)"
Line 166-168: the distinction between antagonistic (defined as +/-, e.g. predator-prey interactions) and mutualistic (+/+, e.g. plant-pollinator) is made here. But what about competitive interactions (-/-) where both partners suffer from an increased abundance of the other partner? It seems this interaction is a bit overlooked here.
Fig 2b: when focusing on mutualistic interactions, how can one distinguish between higher versus lower trophic levels?
Line 263-267: do the authors mean that for single functions, reduced niche overlap is positive, but that for multifunctionality, it is better to have higher niche overlap (functional redundancy)? This should be something that is easy to test.
Line 299-302: the conclusion "Hence, tree biomass cannot approximate the interplay between the multitude of processes that shape forest ecosystem functioning and are included in our study" seems premature. A lack of a relationship between tree biomass and ecosystem multifunctionality could indeed indicate that tree biomass does not affect individual ecosystem functions, but an alternative interpretation is that tree biomass has positive effects on some ecosystem functions, but negative effects on other functions, causing an overall neutral effect on ecosystem multifunctionality. Some follow-up analysis could test this idea, to check whether the current interpretation is correct, or needs to be adjusted.
Line 307: please don't confuse plant productivity with tree biomass, these are different things.
Supplementary Table 7: some of the relationships shown here are intuitive, while others are much more surprising. It would be good to discuss this a bit more.
L 346-349: I find this sentence hard to understand, and would suggest rephrasing
L 384: "To conserve self-sustaining ecosystems…" I am not sure what is meant by this. Are ecosystems with a higher EMF score more "self sustaining" than ecosystems with a lower EMF score? The EMF score is mostly related to biomass stocks and the activity/productivity of organisms, but if these stocks or rates are a bit lower, then does this make an ecosystem less 'self sustaining'?
L 384-386: "effective biodiversity conservation needs to not only focus on rare and threatened species but also target

species interactions and a network structure that supports ecosystem multifunctionality.” How would this work in practice? Or in other words, which management activities or other actions would need to be changed/considered to better target species interactions and a network structure that supports ecosystem multifunctionality?
Table S2: Looking at the ecosystem functions listed here, most are related to either biomass stocks (or proxies thereof) of certain organisms, or their activity/productivity. I think this makes sense, and an implication is that an ecosystem has a higher EMF score if energy stocks or flows are higher. So far, so good. But there are a few ecosystem functions listed that I don’t think fit in very well, as they are neither measures of stocks, nor of fluxes/rates. These are N and P resorption, erosion control and trophobiosis rate. I think it’s confusing to mix these functions into the EMF metric, and would therefore suggest to remove them from the analyses.

Version 1:

Reviewer comments:

Reviewer #1

(Remarks to the Author)
I commend the authors for the improvements made to the manuscript in response to the comments from Reviewer 2 and me. The revisions are thorough, and the authors have addressed my concerns convincingly, providing clear and appropriate justifications where needed. The revisions have strengthened the study's clarity and robustness.

Reviewer #2

(Remarks to the Author)
This is the second manuscript version of this study I have reviewed. Previously, I had several concerns, with some of the more important ones including choices regarding which ecosystem functions were assessed, the discussion of unexpected results and 'overselling' of conservation implications. I am happy to see that all my concerns have been addressed well, and therefore I do not have any remaining concerns.

Open Access This Peer Review File is licensed under a Creative Commons Attribution 4.0 International License, which permits use, sharing, adaptation, distribution and reproduction in any medium or format, as long as you give appropriate credit to the original author(s) and the source, provide a link to the Creative Commons license, and indicate if changes were made.
In cases where reviewers are anonymous, credit should be given to 'Anonymous Referee' and the source.
The images or other third party material in this Peer Review File are included in the article’s Creative Commons license, unless indicated otherwise in a credit line to the material. If material is not included in the article’s Creative Commons license and your intended use is not permitted by statutory regulation or exceeds the permitted use, you will need to obtain permission directly from the copyright holder.
To view a copy of this license, visit https://creativecommons.org/licenses/by/4.0/

Reviewer #1 (Remarks to the Author):

Albert et al. present a compelling and innovative study examining how tree diversity influences ecosystem multifunctionality through the structure of mutualistic and antagonistic interaction networks. The manuscript is well written, methodologically ambitious, and makes an important contribution by integrating concepts of biodiversity–ecosystem functioning with ecological network approaches. The results are clearly presented and supported by strong visualisations. However, I have some suggestions that I believe would improve the clarity, conceptual framing, and robustness of the analyses.

Response: We thank the reviewer for the kind assessment of our study, as well as the insightful suggestions to improve it. We are convinced that the changes made in response improved the quality of our work considerably.

Major Comments

1. Conceptual framing and predictions

The results highlight a set of direct and indirect effects of tree richness on network structure and ecosystem multifunctionality. However, the introduction currently offers limited conceptual background on how and why tree diversity - particularly as primary producers - should influence ecological interactions, network topology, and multifunctionality. Strengthening this section with clearer mechanistic reasoning and explicit hypotheses or predictions would better guide the reader and help position the study within existing theory.

Response R1-1: We appreciate the reviewer's careful reading of our introduction and agree that it indeed largely focuses on how species interactions and network topology could alter ecosystem functioning. In the previous version, we have already argued that species complementarity through niche partitioning is one of the main ways by which primary producer diversity enhances multiple ecosystem functions, and that this can be captured in network structure as trophic complementarity (lines 145-150). Thanks to the reviewer's comment, however, we now realize that the role of species diversity, and producer diversity in particular, should be made more explicit. We therefore decided to expand on how species diversity modifies network topology, competition among species, and how primary producer diversity in particular can modify this:

> *"Species diversity is one of the key variables shaping network structure by increasing the number of interactions[27] and the topological representation of how species compete for shared resources or enemies in networks[14]. Especially when manipulated experimentally, primary producer diversity plays a particularly important role, as it tends to enhance the diversity of other taxa[15,28], for example by providing more diverse resources and habitats[28], and buffering extreme climate events[29]."* (lines 134-139)

2. Joint effects of interaction types

The separate evaluation of antagonistic and mutualistic networks is interesting and informative. Nonetheless, it is not clear why the authors chose not to assess a combined or integrative effect of these networks. Ecological processes often emerge from the interplay among multiple interaction types, and exploring whether or how antagonistic and mutualistic networks jointly mediate biodiversity effects could provide additional insights. At minimum, the rationale for analysing them separately should be clarified.

Response R1-2: We decided to put our main focus on analyzing antagonistic and mutualistic interactions separately mainly for two reasons. First, several previous studies noted that the two types of interactions shape network topology differently. Second, the nature of the interactions creates different links between the functions associated with the two trophic levels. Specifically, we expected that trade-offs between the two trophic levels primarily shape ecosystem functions associated with antagonistic interaction networks, whereas effects should be more tightly associated in mutualistic interaction networks (see also lines 175-178 in Introduction). However, we agree with the reviewer that understanding their joint effects holds additional value for understanding general mediating effects of species interactions on biodiversity-ecosystem multifunctionality relationships. Accordingly, we included such an analysis already in the previous version of the manuscript. Due to the strong similarities with the patterns found for the two interaction types, and to reduce redundancy in our manuscript, we decided to not include the overall analysis in the main text and instead only present it in the supplement (Supplementary Fig. 2). Note that this approach assumes that effects of antagonistic and mutualistic interactions are generalizable, for which we also found support given the clear patterns in the overall trends and our sensitivity analyses (Supplementary Fig. 3). However, based on the reviewer's comment, we have discussed among co-authors how to best test for synergistic effects among network types, including effects between antagonistic and mutualistic interactions. We believe that, in order to properly test such effects, we would require a fundamental redesign of our analytical framework. Additionally, it would shift the focus of our study and introduce an additional layer of complexity that we believe would reduce the accessibility to our study. We therefore decided to instead highlight the joint effects we have already tested more explicitly by making sure to emphasize their generalized nature more prominently (lines 244-247), as well as providing a short outline of our analytical framework and a brief rational for not testing synergistic effects in the methods:

> "*Our main analyses focus on two SEMs, one for antagonistic and one for mutualistic interactions (Fig. 2). We further tested their joint effect with an additional SEM (Supplementary Fig. 2). Note that, to test potential synergistic effects between network and interaction types, a different analytical framework is necessary.*" (lines 557-560)

We further decided to highlight that investigating potential interactive effects between network types would be an important next step to better understand the role species interactions play in biodiversity-ecosystem functioning relationships:

> *"This would also pave the way to empirically establish and generalize potential synergistic interactions between types of networks and interactions, which so far largely focused on the effects on stability and coexistence instead of ecosystem functioning[71,72]."* (lines 425-427)

3. Accounting for hierarchical structure and temporal replication

The experimental design appears hierarchical (plots nested within sites), and sampling spans multiple years (2014–2019). Such structure raises concerns about non-independence among observations. A mixed modelling framework, either linear or generalized, depending on the response variables, would seem more appropriate for handling random site, plot, and temporal effects. In addition, a piecewise SEM approach (e.g., Lefcheck 2016) could offer greater flexibility for integrating causal paths while accounting for this nested design. Clarifying how these dependencies are addressed would enhance confidence in the statistical inference.

Response R1-3: Indeed, our analyses use data collected across several years. Even though we aimed to minimize the temporal spread of our data as much as possible by focusing on assembling data collected in 2018 or as close as possible to that year, we were not able to completely remove it without sacrificing ecosystem functions, network types, and thereby the generality of our work. We, however, shared the

concern of the reviewer, especially for network types, which is why we decided to do a sensitivity analysis where we subsampled network types, and thereby vary the temporal coverage of our data (Supplementary Fig. 3). Given the clear similarities to the findings of the overall trends (Fig. 2), we are confident that the temporal differences should not alter our general conclusions. We have also addressed the issue in our Discussion (lines 250-256).

Regarding the hierarchical structure of our experimental design, the reviewer is correct in noticing that plots (our sampling unit) are nested in two sites. The main reason we decided to not use site as a random effect in our analyses is the common modelling advice to not use random effects with less than 5 factor level (e.g. see https://bbolker.github.io/mixedmodels-misc/glmmFAQ.html#should-i-treat-factor-xxx-as-fixed-or-random). Additionally, based on preliminary analyses where we tested site both as random and fixed effect, we knew that site did not substantially change our findings. This gave us confidence to drop it in the results we presented. However, we acknowledge that we did not make this transparent. We therefore decided to run piecewise SEMs with site as a random effect, which can be found below. The differences to the SEMs without random effects are largely qualitative, with minor changes to path coefficients and a few effects becoming marginally significant. The new SEMs are now presented in the supplementary material (Supplementary Fig. 10, Supplementary Tables 12 & 13) with a reference and short explanation in the manuscript:

> *"Including site as a random effect in our models, using the piecewiseSEM package[105] with linear mixed effect models from the lme4 package[106], led to qualitatively similar results (Supplementary Fig. 10, Supplementary Tables 12 & 13)."* (lines 575-577)

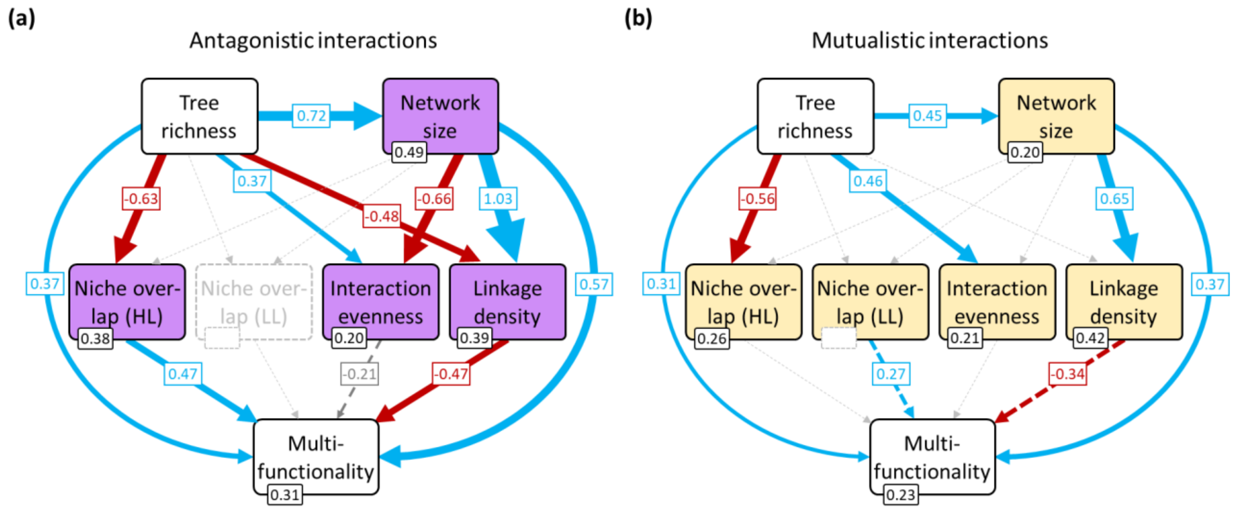

Supplementary Fig. 10: Effects of network structure and tree species richness on ecosystem multifunctionality in (a) antagonistic and (b) mutualistic interaction networks using piecewise structural equation models (SEMs). *SEMs shows tree species richness (i.e. the treatment in the BEF-China experiment) effects on the structure of species interaction networks, and their joint effects on ecosystem multifunctionality. Model structure as in Fig. 2, but including site as a random effect whenever possible. Models are qualitatively the same as the ones presented in Fig. 2. Positive, negative and non-significant standardized path coefficients are indicated in blue, red, and grey, respectively. Marginally significant effects ($p < 0.1$) are indicated as red or blue dashed arrows. R^2-values are displayed at each endogenous variable. Model fit measures: (a) Fisher's C = 0.081, p = 0.96, df = 2; (b) Fisher's C = 26.101, p = 0.097, df = 18. For an overview of model estimates, including covariances between network indices, see Supplementary Tables 12 & 13.*

4. Scope and generality of inference

The experiment includes only two sites, each with multiple subplots. This limited spatial replication constrains the generalizability of the findings. The authors should more clearly discuss the degree to which the observed patterns can be extrapolated beyond the study system, and under what ecological or environmental conditions the proposed mechanisms are expected to hold.

Response R1-4: We thank the reviewer for bringing this issue to our attention. While we agree with the assessment that the scope of our data is somewhat limited, having access to such a wide range of network types and ecosystem functions that were sampled in the same system is rare, even in large coordinated experiments such as ours. However, as we believe the issue to be relevant and worth pointing out, we have now added a short paragraph in our discussion where we specifically highlight that our work is of course limited in scope. There we also provide some potential differences that could be expected when testing our relationships in other systems:

> *"Even though our findings appear robust within our study system, it is important to note that the relationships we observed may differ when investigated somewhere else. While positive effects of biodiversity have been reported from all over the globe and across ecosystems, interaction-based effects can be more context dependent[69]. The importance of specialization for ecosystem multifunctionality we found in our data may be, for example, less relevant in systems where specialization is already high, such as the tropics[70]. The lack of dominance effects found in our study may be a consequence of the comparably high levels of diversity, and may be more pronounced in less diverse systems, for example in boreal or temperate forests. To gain generality on how species interactions mediate biodiversity effects on ecosystem multifunctionality, future research should therefore aim to expand on our work by explicitly testing the investigated relationships across ecosystems."* (lines 401-411)

Minor Suggestion

5. Data transparency and reproducibility

To facilitate reproducibility and improve data accessibility, I recommend including a README file that explains the dataset(s)' structure, variable definitions, and the workflow used for the analyses. This would greatly aid readers attempting to understand or reuse the data and code.

Response R1-5: This is a great suggestion, which we now implemented. Instead of providing a separate file, we have decided to keep everything together and integrate the information in a separate data sheet of the Excel-file. There we also provide a short overview of the project, a brief summary of the analytical approach, and descriptions of the variables used.

Reviewer #2 (Remarks to the Author):

In this study, Albert and co-authors investigated how multritophic interaction networks mediate effects of manipulated tree diversity on ecosystem multifunctionality. Specifically, they hypothesize that because many ecosystem functions are driven by species interactions, and these species interactions depend on (tree or multitrophic) diversity, species interactions should mediate biodiversity-EMF relationships. They tested these predictions based on a large tree biodiversity experiment, where interaction networks as well as various ecosystem function rates were measured.

In general, I am impressed by this study, which relies on a large experiment, huge amounts of data and a nice set of original ideas. At the same time, I do have some concerns which currently still hamper this study.

Response: We thank the reviewer for the thorough assessment of our study, and the insightful comments. We revised our manuscript accordingly, and believe that it considerably enhanced the quality of our work.

First, when looking at the functions that were included to study ecosystem multifunctionality, the majority of the functions were either (proxies of) biomass stocks, or of energy flows. This means that EMF is generally highest in ecosystems with high biomass stocks and flows. So far so good. But there were exceptions of functions that really not fitted into this, and thereby blur the interpretation of the findings. For example, variables like N and P resorption, as well as erosion control are not examples of biomass stocks or flows (even if they are to some extent related, they are fundamentally different), so I do not think it is wise to include these functions when quantifying ecosystem multifunctionality.

Response R2-1: We appreciate the close and critical look at the selection of our variables. Indeed, in our initial conceptualization of the study, we considered erosion, not erosion control, which is much closer to the definition of a process rate we aimed to capture with our multifunctionality index. However, since erosion is an abiotic process in the absence of a biotic safeguard, we decided to inverse it to capture erosion control and thereby the ability of the understory vegetation, tree roots, and the biocrust to conserve the soil they rely on. Similarly, we understood a higher N and P resorption as an increased efficiency of the trees of our forest ecosystem, which use their resources more completely. While we believe that both, erosion control and resorption, are important biotic processes that crucially add to the functioning of the system, we agree that they may not fit a strict definition of ecosystem functioning. We therefore decided to test how much they affect our multifunctionality index and found that correlations between multifunctionality with and without the three variables in question are very high (r = 0.99), which gives us confidence that our findings are robust to the inclusion of resorption and erosion control. We now included the correlation in Supplementary Fig. 9 and mention the additional analyses in the text:

> *"Note that erosion control as well as nitrogen and phosphorus resorption represent important biotic processes (i.e. the ability of the understory vegetation, tree roots, and the biocrust to conserve the soil they rely on, as well as the ability of trees to use their resources more completely) but may deviate from this strict definition. Their exclusion, however, barely affect ecosystem multifunctionality (Supplementary Fig. 9)."* (lines 447-451)

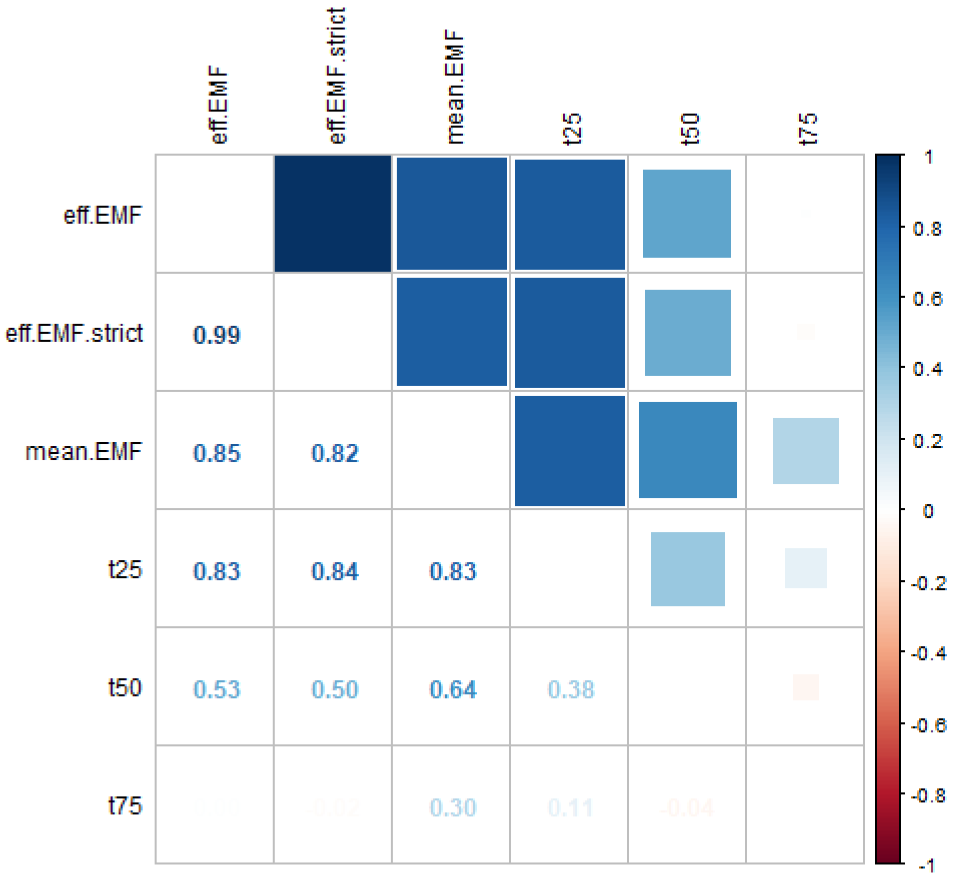

Supplementary Fig. 9: Correlations between different ecosystem multifunctionality indices. *Indices shown are: effective ecosystem multifunctionality (eff.EMF; see Byrnes et al. 2023 and Methods for further information), as used for the analyses in this study; effective ecosystem multifunctionality excluding erosion control as well as nitrogen and phosphorus resorption to fit a strict definition of ecosystem functions (eff.EMF.strict); average ecosystem multifunctionality (mean.EMF); threshold-based ecosystem multifunctionality, at 25%, 50%, and 75% thresholds (t25, t50, t75). Pearson correlation coefficient is shown in lower triangle. Square size in upper triangle indicates absolute value of correlation coefficient (blue: positive; red: negative).*

Second, when looking at the more detailed results (on individual ecosystem functions) that are a bit hidden in Supplementary Table 7, I found that some observed relationships were rather surprising. For example, spider-prey interactions seem rather predictive of fungal fruitbody numbers, while understory biomass was most related to spider-prey interactions (based on spiders that were active in aboveground tree branches). It would be good to discuss the presence of unexpected relationships a bit, even though this discussion should mostly focus on a meta-level, as one can’t possibly discuss everything.

Response R2-2: We agree with the reviewer that there are some unexpected relationships in the data, which we have already highlighted for tree biomass in the previous version (lines 296-300) and which we consider to indicate the complexity of ecological interactions that warrant a multitrophic perspective in analyzing ecosystem multifunctionality. We also agree that it is worth mentioning those somewhat unexpected associations more generally and discuss potential reasons for those associations, namely similarities in network structure across network types, and potential cascading effects and higher-order

interactions. We decided to illustrate these potential effects on the example of spider-prey networks and fruitbody numbers, as we found it particularly striking:

> *"Our findings on tree productivity therefore also demonstrate that the effects of single bottom-up processes are limited. This finds further confirmation in the significant association between network types and not directly associated functions (Supplementary Table 7). For example, the structure of spider-prey interaction networks shows significant effects on the number of fungal fruitbodies involved in wood decomposition processes. While such associations can result from a network structure that is consistent across types of networks, it may also highlight cascading effects and higher-order interactions[42]. Spiders hunting decomposers can, for example, alter decomposition processes and nutrient availability[43,59], which could in turn lead to the observed effects on fungal fruitbody numbers. By utilizing network analyses to investigate drivers of biodiversity–ecosystem functioning relationships, our findings therefore indicate that multiple processes, including species interactions and their potential cascading effects, act in concert to determine ecosystem multifunctionality."* (lines 313-324)

The authors also tried to sell their paper a bit as informing conservation. For example, at the end of the abstract, the authors write "These findings emphasize that to effectively safeguard ecosystem service provisioning, ecosystem management and biodiversity conservation not only need to account for biodiversity changes at multiple trophic levels, but also explicitly for how species interact among each other." It is quite popular in ecological papers to claim that findings have applied value for nature management, but a statement like this feels a bit empty if not any concrete examples (also not in the main text) are given. So if the authors believe in this statement, then I would also expect them to really work this argument out, and give concrete examples (so: how can e.g. forest managers adjust their management in order to stimulate species interactions that promote ecosystem multifunctionality? And is it likely that they can be convinced to do so, or would they have other priorities, even if they do care about biodiversity conservation). Otherwise, it would be better to remove sentences like these.

Response R2-3: While we agree with the assessment of previous studies that conservation efforts should consider the species interaction networks (e.g. Tylianakis et al. 2010, Harvey et al. 2017), we realized thanks to the reviewer's comment that our call for action remains somewhat superficial. In an attempt to think through and read up on the issue, we also realized that the effective implementation of network-based conservation measures requires better monitoring schemes that can rapidly assess networks to begin with. This would then also allow to close important knowledge gaps, for example by allowing to test if existing conservation measures may already support desirable network characteristics. To reflect this change of focus, we have rephrased the end of our abstract accordingly:

> *"These findings emphasize that ecosystem service provisioning does not only rely on biodiversity at multiple trophic levels, but to a similar degree on how species interact among each other."* (lines 112-114)

We also decided to tone down the call for action in the end of our discussion by removing "conservation" from the chapter's heading, and by highlighting future research direction that would make network-based conservation efforts feasible:

> *"Making the implementation of network-based conservation measures feasible, however, requires approaches to rapidly assess species interaction networks[68]. With modern methods for assessing species and communities becoming increasingly accessible, developing and challenging predictive approaches to determine species interactions therefore remain the main hurdle in this endeavor."* (lines 396-400)

References:

Tylianakis, J. M., Laliberté, E., Nielsen, A. & Bascompte, J. Conservation of species interaction networks. Biol Conserv 143, 2270–2279 (2010).

Harvey, E., Gounand, I., Ward, C. L. & Altermatt, F. Bridging ecology and conservation: from ecological networks to ecosystem function. J Appl Ecol 54, 371–379 (2017).

I also have several more specific comments, listed below.

L120-122: the high number of dependent clauses makes this sentence rather difficult to understand

Response R2-4: We have now simplified the sentence:

> *"As a consequence, critical ecosystem functions (i.e. ecosystem process rates such as productivity, herbivory, and nutrient cycling)[8], many of which directly result from species interactions[9], are at risk."* (lines 120-122)

L129-130: "limits our ability to accurately predict the consequences of global biodiversity": I find the focus on global biodiversity a bit strange, because species interactions seem mostly relevant at local scales (plants/animals need to be sufficiently close to each other to interact), but less so at very large (or even global) scales.

Response R2-5: Thank you for pointing this out. We now realize that the formulation was indeed misleading. What we intended to convey was biodiversity change that we see everywhere across the globe. To avoid this confusion, we have now removed "global" from the sentence.

Line 135: "to accurately predict the consequences of global biodiversity": would be nice to add an argument on why this is the case

Response R2-6: We realize that accurate predictions might be possible, even without understanding the driving mechanisms. We therefore decided to change this section accordingly:

> *"[…] the incomplete understanding of the driving forces behind biodiversity-mediated alterations to ecosystem functioning limits our ability to make process-based predictions of the consequences of biodiversity change […]"* (lines 127-129)

L 139: "biodiversity" should be replaced by "biodiversity-ecosystem functioning"

Response R2-7: We appreciate this suggestion as it helps us to avoid the otherwise necessary definition of the somewhat ambiguous term "biodiversity mechanisms". Accordingly, we now talk about "biodiversity-ecosystem functioning (BEF) mechanisms" instead (also throughout the manuscript) and removed the now unnecessary definition.

L 144-146: I found this mechanism not so well explained yet. What is meant by "complementarity in species interactions"? Can you give an example? For example, are competitive and trophic interactions complementary to each other? And what does complementarity mean in this context?

Response R2-8: What we mean with a complementarity in species interactions are differences in interaction partners. For a consumer species, this would be a complementarity in resource-use, for a

resource species, it is a complementarity in enemies. As both perspectives reflect the presence or absence of an indirect competitive interactions (exploitative or apparent, sensu Holt 1977), a higher complementarity would mean a lower competition and vice versa. We now clarify this in the text:

> *"[…] trophic complementarity (i.e. complementarity in species interactions due to different interaction partners) […]"* (lines 147-148)

References:

Holt, R. D. Predation, Apparent Competition, and the Structure of Prey Communities. Theor Popul Biol 12, 197–229 (1977).

L 151-152: it would be good to explain a bit more what is meant by "the evenness distribution of species interactions (i.e. interaction evenness)"

Response R2-9: We have now rephrased the section to make interaction evenness easier to understand:

> *"[…] it can be important to also take into account whether species interactions within a network are dominated by few or are evenly distributed among species (i.e. interaction evenness)."* (lines 154-156)

Line 166-168: the distinction between antagonistic (defined as +/-, e.g. predator-prey interactions) and mutualistic (+/+, e.g. plant-pollinator) is made here. But what about competitive interactions (-/-) where both partners suffer from an increased abundance of the other partner? It seems this interaction is a bit overlooked here.

Response R2-10: While we do not deny that competition can play a major role in ecosystems, including in our experimental system, we believe the nature of competition fundamentally differs from the antagonistic and mutualistic interactions our study focuses on. Specifically, the interaction networks we focus on span two trophic levels, whereas competitive interactions are usually constrained within trophic levels. Additionally, competition is usually indirect, as it is mediated by a shared resource or enemy (i.e. exploitative and apparent competition sensu Holt 1977). In this way, our networks actually include these competition motifs. We now clarify this in the methods when we introduce the sampled networks:

> *"In contrast to competition networks that are capturing interactions within trophic groups, antagonistic and mutualistic networks focus on the interactions between two distinct trophic groups, with competition being an implicit property[93]."* (lines 456-458)

References:

Holt, R. D. Predation, Apparent Competition, and the Structure of Prey Communities. Theor Popul Biol 12, 197–229 (1977).

Fig 2b: when focusing on mutualistic interactions, how can one distinguish between higher versus lower trophic levels?

Response R2-11: Each of the mutualistic networks has a clear primary resource provider, which we defined as the lower trophic level. In most cases these are trees that provide resources to the mycorrhizal and saprophytic fungi, as well as to the endophytic bacteria. Additionally, we have Hemiptera species providing resources to the ants. In return, the higher trophic levels do not necessarily offer resources. Even though they often improve resource acquisition, they are not strictly necessary for the survival of the lower trophic level. While we agree that distinguishing higher and lower trophic levels in mutualistic interaction

networks is less straightforward than in antagonistic networks, we still believe that it is possible. To make this clearer, we now added some additional information in the methods:

> *"We defined the lower trophic level as the trophic level that primarily provides resources, and the higher trophic level as the one that utilizes the resources."* (lines 515-517)

Line 263-267: do the authors mean that for single functions, reduced niche overlap is positive, but that for multifunctionality, it is better to have higher niche overlap (functional redundancy)? This should be something that is easy to test.

Response R2-12: Thanks to the reviewer's comment, we realized that the way we discussed effects of linkage density and niche overlap were too much intertwined, leading to a misunderstanding of our argument, which was not related to niche overlap but linkage density. We have now reworked the section of the manuscript to separate the discussion of both network indices more clearly:

> *"While the structure of multitrophic species interaction networks is not suited to investigate the role of facilitation, our findings indicate that niche differentiation leads to an increase in the complementarity among species, as tree species richness reduces the niche overlap of higher trophic levels and favours species with narrower niches (i.e. specialists, low linkage density; Fig. 2). The latter also supports ecosystem multifunctionally, and thereby aligns with patterns expected from niche differentiation. So far, most of the evidence for drivers of biodiversity effects comes from studies of individual ecosystem functions[19,30,46]. While a few species can suffice to provide these functions, substantially more species are required to maintain ecosystem multifunctionality[47,48]. Hence, species may require relatively narrow niches (i.e. specialists, low linkage density) to make unique contributions to ecosystem multifunctionality. However, while we found positive effects of narrow niches (i.e. low linkage density), a reduced niche overlap had a negative rather than the expected positive effect in antagonistic networks (Fig. 2a). In mutualistic networks, similar effects were found for niche overlap between species at the lower trophic level (Fig. 2b). Hence, our findings suggest that there is an additional way by which species occupying similar niches (i.e. high niche overlap) collectively contribute to ecosystem multifunctionality."* (lines 259-274)

Line 299-302: the conclusion "Hence, tree biomass cannot approximate the interplay between the multitude of processes that shape forest ecosystem functioning and are included in our study" seems premature. A lack of a relationship between tree biomass and ecosystem multifunctionality could indeed indicate that tree biomass does not affect individual ecosystem functions, but an alternative interpretation is that tree biomass has positive effects on some ecosystem functions, but negative effects on other functions, causing an overall neutral effect on ecosystem multifunctionality. Some follow-up analysis could test this idea, to check whether the current interpretation is correct, or needs to be adjusted.

Response R2-13: This is a very good observation we did not fully explore previously. From Supplementary Figs. 4 & 5, we could already see that tree biomass shows relatively distinct trends with the different network indices when compared to other functions. However, to test the association between tree biomass and the other functions further, we conducted a correlation analysis, where we found that biomass indeed shows significant (positive or negative) correlations with only a few functions, whereas with most functions correlations were non-significant (Supplementary Fig. 7, also below). To better reflect this, we now changed our previous statement to:

> *"Hence, tree biomass does not emerge as the main driver of the interplay between the multitude of processes that shape forest ecosystem functioning and are included in our study. This is also apparent*

from the largely non-significant correlations of tree biomass with other ecosystem functions (Supplementary Fig. 7)." (lines 305-308)

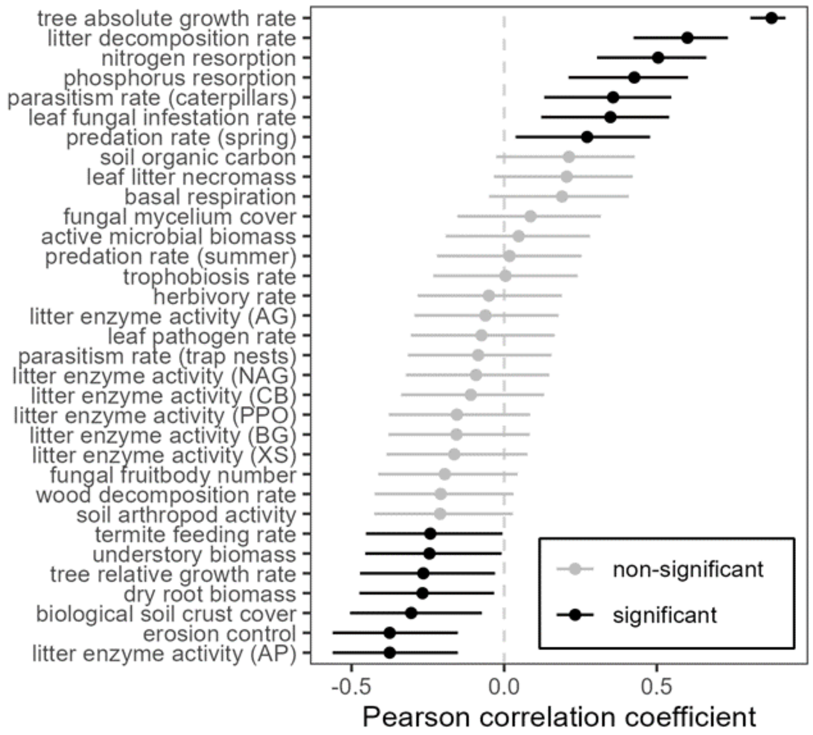

Supplementary Fig. 7: Correlation between tree biomass and the other ecosystem functions included in our study. *Points show Pearson correlation coefficient and error bars indicate confidence interval. Significant correlations in black, non-significant correlations in grey.*

Line 307: please don't confuse plant productivity with tree biomass, these are different things.

Response R2-14: We now correctly refer to tree biomass (now in line 312).

Supplementary Table 7: some of the relationships shown here are intuitive, while others are much more surprising. It would be good to discuss this a bit more.

Response R2-15: Please see our response to your second major comment.

L 346-349: I find this sentence hard to understand, and would suggest rephrasing

Response R2-16: The have rephrased the sentence, which now reads:

"Linkage density had negative effects on multifunctionality and thus indicates the importance of specialized interactions, which also created a positive indirect effect of tree species richness on ecosystem multifunctionality." (lines 359-361)

L 384: "To conserve self-sustaining ecosystems…" I am not sure what is meant by this. Are ecosystems with a higher EMF score more "self sustaining" than ecosystems with a lower EMF score? The EMF score is

mostly related to biomass stocks and the activity/productivity of organisms, but if these stocks or rates are a bit lower, then does this make an ecosystem less 'self sustaining'?

Response R2-17: This section was now removed in response to the reviewer's third major comment.

L 384-386: "effective biodiversity conservation needs to not only focus on rare and threatened species but also target species interactions and a network structure that supports ecosystem multifunctionality." How would this work in practice? Or in other words, which management activities or other actions would need to be changed/considered to better target species interactions and a network structure that supports ecosystem multifunctionality?

Response R2-18: This section was now removed in response to the reviewer's third major comment.

Table S2: Looking at the ecosystem functions listed here, most are related to either biomass stocks (or proxies thereof) of certain organisms, or their activity/productivity. I think this makes sense, and an implication is that an ecosystem has a higher EMF score if energy stocks or flows are higher. So far, so good. But there are a few ecosystem functions listed that I don't think fit in very well, as they are neither measures of stocks, nor of fluxes/rates. These are N and P resorption, erosion control and trophobiosis rate. I think it's confusing to mix these functions into the EMF metric, and would therefore suggest to remove them from the analyses.

Response R2-19: Please see our response to your first major comment. Note that we have excluded trophobiosis rates from the added sensitivity test, because trophobiosis rates reflect not just the protection of Hemiptera by ants, but also the consumption of honeydew by ants, hence capture a flux. To make this more clear, we have now added a short explanation to the Supplementary Table 1:

> *"Trophobiosis rates (i.e. ants protecting Hemiptera in exchange for honeydew) […]"*
